# Temperature-Related Variations in Physical Performance During Elite Soccer Matches

**DOI:** 10.3390/sports12120341

**Published:** 2024-12-07

**Authors:** Vladimir Pavlinovic, Ryland Morgans, Toni Modric

**Affiliations:** 1 Faculty of Kinesiology, University of Split, 21000 Split, Croatia; vladimirpavlinovic@gmail.com; 2 School of Sport and Health Sciences, Cardiff Metropolitan University, Cardiff CF23 6XD, UK; rmorgans@cardiffmet.ac.uk; 3 High Performance Sport Center, Croatian Olympic Committee, 10000 Zagreb, Croatia

**Keywords:** running performance, ambient temperature, soccer, environmental factors

## Abstract

The aim of this study was to examine the differences in match running performance (MRP) according to the ambient temperature during UEFA Champions League (UCL) matches. Data were collected using an optical tracking system from all teams (*n* = 32) in all UCL matches (*n* = 125) during the 2022/23 season, and classified according to the ambient temperature at which matches were played: <5 °C, 6–10 °C, 11–20 °C, and >21 °C. The results revealed the following: (i) less total distance was covered in matches played at ≥21 °C compared to the matches played at 6–10 °C (d = 0.58), (ii) less high-speed running and high-intensity running were covered in matches played at ≥21 °C compared to the matches played at 11–20 °C (d = 0.54 and 0.43, respectively), 6–10 °C (d = 0.89 and 0.8, respectively), and ≤5 °C (d = 0.62 and 0.57, respectively), and (iii) less sprinting was covered in matches played at ≥21 °C compared to the matches played at 6–10 °C (d = 0.22). These findings indicated the significant differences in MRP when UCL matches were played at different ambient temperatures, with notable reductions in overall and high-intensity efforts in warmer conditions.

## 1. Introduction

Soccer is a complex sport characterized by high physical demands, including covering large total distances and performing repeated high-intensity sprints and explosive efforts [1,2]. The most common technique to evaluate physical demands during soccer match-play is an analysis of the players’ match running performance (MRP) [3,4]. The MRP typically includes total distance covered, distance covered in different speed zones, accelerations, and decelerations [5,6]. The data on MRP are used by sports scientists primarily to help practitioners in decision-making processes for structuring the elements of training and subsequent match preparation [7]. However, soccer is subject to various situational and environmental factors that affect MRP [8,9,10], hampering its interpretation and application in practice [11]. Specifically, situational factors within a game, such as a match outcome, match location, and opponent quality have been evaluated with differing results [12,13,14,15]. Because of physiological constraints when working in certain settings, environmental elements such as ambient temperature have also been shown to have an impact on MRP [16,17]. 

Specifically, when working in a hot environment, the body’s thermoregulatory system activates mechanisms such as increased sweating to dissipate the heat [18]. This can cause electrolyte imbalances and dehydration, which can harm the heart and lower one’s ability to do continuous physical exertion [19]. Also, working in a hot environment raises metabolic stress, prompting faster glycogen breakdown and a higher reliance on anaerobic energy sources, which can lead to early fatigue [20,21]. Conversely, prolonged exposure to cold conditions can affect the body’s shivering response and cause peripheral blood vessels to constrict, potentially leading to quicker fatigue and diminished exercise performance over time [19,22].

Research suggests a decline in MRP when temperatures exceed 29 °C [23]. Reports indicate a significant increase in low-intensity demands and exercise time below 85% of maximal heart rate compared to moderate temperatures [23]. Consistent with these findings, another study reported significant effects of hot temperatures on running speed, high-speed runs, and high-intensity and explosive distance covered [24]. When considering FIFA 2014 games, the comparison between low and high temperatures did not reveal significant differences in total distance covered [25]. However, it did show that more intense actions such as the number of sprints were decreased in hotter temperatures, as well as the distance covered at high intensity [26]. Examining the impact of cold temperatures, a study involving professional soccer players generally suggested that physical performance in professional soccer does not decrease in colder conditions [26]. This contrasts with another study which found that at sub-zero temperatures, every 1 °C decrease reduced the team’s sprint distance by 19.2 m [27]. However, the study also revealed that total, running, and high-speed running distances showed no significant differences across temperatures up to 10 °C [27].

While ample research exists regarding the influence of ambient temperature on the MRP of soccer players [3,4], this body of research has yet to explore multilevel mixed modeling as an alternative approach for examining the results. This approach offers the capability to assess the independent effects of a variable on an outcome while accounting for all other variables that could affect results (i.e., situational factors), allowing the true differences to be elucidated. Furthermore, although various competitions have been studied [8,28], no studies so far have analyzed the entire number of matches within high-elite competitions such as the UEFA Champions League (UCL) in the context of ambient temperature. This analysis has the potential to enhance the generalizability of evidence while providing a benchmark for elite practitioners. Thus, this study contributes to the existing body of knowledge by addressing two critical gaps: (1) the application of advanced statistical modeling to account for situational factors and (2) the comprehensive analysis of all UCL matches to provide insights specific to the highest levels of elite soccer. Therefore, the aim of the study was to examine the differences in MRP according to the ambient temperature during the UCL matches while controlling for situational factors. 

## 2. Materials and Methods

### 2.1. Design

An observational study design was employed to examine the influence of ambient temperature on MRP during matches in the 2022/23 UCL season.

### 2.2. The Match Data 

The MRP data were collected using an optical tracking system (Player & Ball Tracking System, Hawk-Eye Innovations Limited, Basingstoke, England). The system’s reliability has previously been tested and reported [29]. Measurements of ambient temperature prior to kick-off in matches are systematically taken by match-day officials belonging to the UEFA. For the purpose of this study, the measurements were subsequently obtained from the official UEFA match reports. As suggested previously [26,27], the following temperature classification was employed: ≤5 °C = very cold, 6–10 °C = cold, 11–20 °C = moderate, and ≥21 °C = warm.

### 2.3. Match Analysis 

The MRP was obtained from all teams (*n* = 32) playing in all matches (*n* = 125) matches of the UCL in the 2022/23 season. Three matches were initially excluded due to poor data quality, resulting in a final sample of 122 matches. For each match, two observations (one per team) were analyzed, yielding a total of 244 team observations for analysis. These observations were classified into four groups according to the ambient temperature at which matches were played: <5 °C (8 observations), 6–10 °C (28 observations), 11–20 °C (144 observations), and ≥21 °C (66 observations). To avoid the influence of situational factors which were shown to influence MRP [8,9,10], match outcome and location, quality of opponent, ball-in-play time, and red cards were noted in all matches. All data were anonymized in accordance with the principles of the Declaration of Helsinki to ensure confidentiality. The investigation was approved by the local university ethics board. The descriptive statistics of MRP according to the ambient temperature is presented in Table 1.

### 2.4. Variables

Dependent variables (i.e., MRP) included total distance covered (TD) (m), low-intensity running (LIR) (m) (≤15 km/h), moderate-intensity running (MIR) (m) (15–20 km/h), high-intensity running (HSR) (m) (20–25 km/h), sprinting (SPR) (m) (≥25 km/h), and high-intensity running (HIR) (m) (≥20 km/h) [30]. The independent variables were a four-level categorical variable “ambient temperature”, the three-level categorical variables “match outcome”, two-level categorical variables “match location”, and “red card”, and the continuous variables “opponent quality” and “ball-in-play”. Match outcome was categorized as win, draw, or loss, while match location was recorded as either home or away. The presence of a red card was noted with a “yes” or “no” based on whether a red card was issued during the match. Opponent quality was evaluated using the UEFA 5-year club coefficients. Ball-in-play time, as defined by the data provider, refers to the duration when the ball is actively possessed by players or available for either team to contest. Periods when the referee stops play are considered out of play and do not count towards ball-in-play time [31].

### 2.5. Statistical Analysis 

The differences in MRP according to ambient temperature were examined using linear mixed models (LMM). Matches and teams were included as random effects. A “step-up” model construction strategy was employed, beginning with the null model. Independent variables were included if they improved model information criteria and showed statistical significance (*p* ≤ 0.05) over the previous model. Model selection relied on the Akaike information criterion (AIC), with lower values indicating a better fit, and on the chi-square likelihood ratio test. Specifically, models were compared by calculating the difference in log-likelihood values between the new and previous models, with degrees of freedom based on the difference in parameters. The final models were selected based on improved AIC, log-likelihood, and significant variable effects. Model fit was evaluated using residuals versus fitted plots and Q–Q plots [32,33]. T-statistics from the LMM were transformed into effect sizes (Cohen’s d) with corresponding 95% confidence intervals (CIs). These effect sizes were interpreted as follows: less than 0.2 as trivial, 0.2–0.5 as small, 0.5–0.8 as medium, and greater than 0.8 as large (8). All analyses were conducted using SPSS software, version 25.0 for Windows (IBM, Armonk, NY, USA), with a significance level set at 0.05.

## 3. Results

Visual inspections of residual plots for models 1, 2, 4, 5, and 6 showed no noticeable deviations from homoscedasticity or normality, with Q–Q plots indicating a good data fit. However, model 3 did not effectively represent the data structure, so the effect of ambient temperature on MIR was not further analyzed.

Examining the explained variance attributed to fixed effects alone, the models showed marginal r^2^ values as follows: 0.22 for TD, 0.56 for LIR, 0.09 for HSR, 0.08 for SPR, and 0.10 for HIR. When including both fixed and random effects, conditional r^2^ values were 0.95 for TD and LIR, 0.90 for HSR, 0.92 for SPR, and 0.94 for HIR (Table 2 and Table 3).

Table 2 shows that ambient temperature influenced TD (f = 3.34, *p* < 0.001). Specifically, less TD was covered in matches played at ≥21 °C (108.96 km) compared to the matches played at 11–20 °C (110.35 km, trivial ES) and 6–10 °C (112.13 km, medium ES). As covariates, match outcome (f = 18.46, *p* < 0.001), ball-in-play (f = 23.89, *p* < 0.001), and red cards (f = 9.34, *p* = 0.003) influenced TD. No effect of ambient temperature on LIR was found (f = 0.36, *p* = 0.758).

Table 3 shows that ambient temperature influenced HSR (f = 8.91, *p* < 0.001), SPR (f = 9.01, *p* < 0.001), and HIR (f = 7.26, *p* < 0.001). Specifically, less HSR was covered in matches played at ≥21 °C (6.37 km) compared to the matches played at 11–20 °C (6.73 km, medium ES), 6–10 °C (7.3 km, large ES), and ≤5 °C (7.4 km, medium ES). Also, less SPR was covered in matches played at ≥21 °C (2.20 km) compared to the matches played at 6–10 °C (2.57 km, medium ES). Finally, less HIR was covered in matches played at ≥21 °C (8.67 km) compared to the matches played at 11–20 °C (9.1 km, small ES), 6–10 °C (9.86 km, large ES), and ≤5 °C (10.03 km, medium ES). As covariates, match location had an influence on SPR (f = 21.77, *p* < 0.001) and HIR (f = 13.57, *p* < 0.001).

## 4. Discussion

The aim of this study was to examine differences in MRP according to the ambient temperature during UCL matches while controlling statistically for the influence of situational factors. The results revealed decreased TD, HSR, SPR, and HIR in matches played in a warm environment. On the other hand, a similar distance covered at a lower speed was found irrespective of the ambient temperature. These findings may be of great interest to support practitioners entrusted with safeguarding the players’ well-being.

Previous studies examining the differences in MRP according to the ambient temperature in soccer mostly examined findings from a single country [27,34] or a single team [24]. Such findings were undoubtedly influenced by the specificities (i.e., geographical, cultural, historical, and/or social) of observed competitions and teams [35]. There are also several studies using limited samples [24] or data that can be currently considered outdated [26] due to the tremendous increases in MRP in the last decade [36]. Finally, studies have rarely controlled for the situational factors that have been repeatedly demonstrated to influence physical performance during soccer matches [8]. Consequently, it has been difficult to determine the actual differences in MRP according to the ambient temperature. To address the relevant literature gaps, the current study examined these differences by investigating teams playing in multiple countries while also controlling for several potential situational factors. The results showed that less TD was covered in UCL matches played at ≥21 °C compared to the matches played at 6–10 °C (medium ES). In addition, less HSR and HIR were covered in matches played at ≥21 °C compared to the matches played at 11–20 °C (medium and small ES, respectively), 6–10 °C (both large ES), and ≤5 °C (both medium ES). Also, less SPR was covered in matches played at ≥21 °C compared to the matches played at 6–10 °C (medium ES). 

These findings clearly indicated a decrease in overall and intensive efforts in warm compared to cold environments. It has been speculated that this may be the result of pacing strategies for players to preserve their technical skills and peak running speed [25]. However, we are of the opinion that the unique feature of the observed competition (i.e., UCL) has affected our findings. As a top soccer club tournament involving the world’s best players, the UCL is distinguished by highly professional and driven athletes who maintain consistently high running performance regardless of opponent or match outcome [12]. This may indicate a low reliance on pacing tactics. Therefore, decreased overall and intensive efforts in warm compared to cold environments may rather be a consequence of the body’s efforts to dissipate the heat. 

Specifically, when exposed to high temperatures, the body’s thermoregulatory systems activate, causing reactions such as increased perspiration to aid heat [18]. Excessive sweating can cause dehydration and electrolyte imbalances, impairing cardiovascular homeostasis [19]. This might reduce the capacity for prolonged physical exertion, resulting in reduced overall efforts. Increased temperatures, on the other hand, worsen metabolic strain during exercise, resulting in rapid glycogen depletion and increased dependence on anaerobic energy pathways, ultimately leading to early exhaustion [20,21]. As the anaerobic system is associated with high-intensity efforts [37], such overload most likely resulted in reduced intensive efforts.

Our findings are generally consistent with previous studies investigating the French League 1, Russian Premier League, and German Bundesliga 1 [26,27,34,38]. However, these studies lacked data related to situational variables that have been repeatedly demonstrated to affect MRP in soccer [8]. Failure to do so brings into question any conclusions drawn from the data. Therefore, in the current study, the differences in MRP according to the ambient temperature were investigated while accounting for match outcome and location, quality of opposition, ball-in-play time, and red cards. This approach strengthens the true impact of ambient temperature on MRP in elite soccer players while eliminating the likelihood that the findings are merely due to natural match-to-match variability [39]. Indeed, our results demonstrated a substantial impact of match outcome, effective playing time, and red cards on TD, whereas match location had an influence on SPR and HIR.

It is particularly important to highlight that high-intensity efforts (i.e., HSR, SPR, and HIR), a metric commonly deemed critical to match outcome [40,41], in UCL matches played in warm environments were reduced by approximately 15%. Therefore, when confronted with high ambient temperature (over 20 °C), (i) coaches should consider adopting alternative strategies rather than striving to maintain HIR levels equivalent to those in colder environments, and (ii) practitioners entrusted with safeguarding the players’ well-being should consider implementing structured hydration plans before, during, and after matches. When competing in the UCL, this is paramount for sustaining players’ performance throughout a match and reducing the risk of injury or heat-related illnesses. The practical relevance of these conclusions is confirmed by the medium-to-large effect sizes [40]. 

Several limitations should be noted when interpreting the findings of this study. Firstly, the potential temperature variations throughout the matches were not taken into account, which would have affected the results. Secondly, match observations were classified into four groups according to the ambient temperature at which matches were played. However, groups were unequal in their sample sizes and such discrepancy likely influenced the findings. Thirdly, although the most influential factors that can affect MRP were considered, future studies should control for other match-related factors that may be impact results such as stage effect (i.e., group vs. knockout), playing position or team formation. For a more comprehensive understanding of environmental conditions effects, additional environmental factors such as relative humidity, atmospheric pressure, wind, and precipitation should be investigated in future studies. Also, there should be some attempt to account for differences in accelerations and decelerations according to ambient temperature. This may enable a more detailed understating of the physical demands required when playing in specific environmental conditions. 

## 5. Conclusions

As far as we know, this study represents the inaugural large-scale investigation aimed at examining how the ambient temperature impacts the MRP of elite soccer teams, enhancing the generalizability of evidence while providing a benchmark for elite practitioners competing in UCL. The results indicated significant differences in MRP when matches were played at different ambient temperatures, with significant reductions in overall (e.g., TD) and high-intensity efforts (e.g., HSR, SPR, HIR) in warmer conditions (≥21 °C). It is therefore imperative for practitioners to account for ambient temperature when preparing for UCL matches. Specifically, when ambient temperature is forecasted to be over 20 °C, (i) soccer coaches could explore using different playing styles to achieve tactical goals while reducing physical strain, (ii) medical staff could consider structured hydration before the match and cooling techniques during half-time, and (iii) strength and conditioning coaches could reduce external training loads in the trainings preceding such matches. This is especially important for teams from northern Europe accustomed to cooler temperatures, which might experience more significant MRP declines when playing in warmer conditions.

## Figures and Tables

**Table 1:** Match running performance at different ambient temperatures (data are given as mean ± SE).

	≤5 °C	6–10 °C	11–20 °C	≥21 °C
Total distance (km)	111.67 ± 1.73	112.13 ± 1.09	110.35 ± 0.7	108.96 ± 0.83
Low-intensity running (km)	83.37 ± 1.2	84.42 ± 0.74	84.59 ± 0.44	84.56 ± 0.54
Moderate-intensity running (km)	18.39 ± 0.61	18.03 ± 0.38	16.87 ± 0.24	15.88 ± 0.28
High-speed running (km)	7.4 ± 0.31	7.3 ± 0.19	6.73 ± 0.11	6.37 ± 0.14
Sprinting (km)	2.62 ± 0.18	2.57 ± 0.1	2.37 ± 0.05	2.29 ± 0.07
High-intensity running (km)	10.03 ± 0.45	9.86 ± 0.27	9.1 ± 0.15	8.67 ± 0.19

**Table 2:** The effect of ambient temperature on total distance and distances covered at lower speeds while controlling for situational factors.

	Total Distance (Model 1)			Low-Intensity Running (Model 2)			Moderate-Intensity Running (Model 3) *		
Fixed Effects	Coef (SE)	95%CI	Effect Size	Coef (SE)	95% CI	Effect Size	Coef (SE)	95%CI	Effect Size
Intercept	86.76 (4.24)	78.37–95.15		63.24 (2.99)	57.33–69.16		-	-	
TEMP: ≤5 °C	2.71 (1.75)	−0.76–6.19	0.15 (−0.04–0.34)	−1.19 (1.24)	−3.64–1.27	−0.19 (−0.57–0.2)	-	-	-
TEMP: 6–10 °C	3.17 (1.07)	1.04–5.29	0.58 (0.19–0.97)	−0.14 (0.76)	−1.64–1.36	−0.04 (−0.42–0.35)	-	-	-
TEMP: 11–20 °C	1.39 (0.69)	0.02–2.75	0.01 (−0.37–0.39)	0.03 (0.49)	−0.93–0.99	−0.11 (−0.5–0.27)	-	-	-
MO: Loss	−1.73 (0.33)	−2.38–−1.09	−1 (−1.4–−0.61)	−1.58 (0.25)	−2.09–−1.08	−1.17 (−1.56–−0.77)	-	-	-
MO: Draw	1.42 (0.78)	−0.14–2.97	0.35 (−0.03–0.73)	1.16 (0.56)	0.06–2.26	0.4 (0.02–0.78)	-	-	-
ML: Home	-	-	-	-	-	-	-	-	-
OQ	-	-	-	-	-	-	-	-	-
BP	0.36 (0.07)	0.21–0.5	0.94 (0.54–1.33)	0.35 (0.05)	0.25–0.45	1.28 (0.87–1.69)	-	-	-
RC: No	2.55 (0.83)	0.9–4.21	0.6 (0.21–1)	1.82 (0.59)	0.65–2.99	0.6 (0.21–0.99)	-	-	-
Random effects	Coef (SE)	95%CI	*p*	Coef (SE)	95%CI	*p*	Coef (SE)	95%CI	*p*
Match	8.3 (1.49)	5.84–11.81	<0.001	3.92 (0.73)	2.72–5.65	<0.001	-	-	-
Team	8.5 (2.48)	4.79–15.05	0.001	2.65 (0.83)	1.44–4.89	0.001	-	-	-
Residuals	3.17 (0.47)	2.38–4.23	<0.001	2.04 (0.29)	1.54–2.7	<0.001	-	-	-
R^2^_marginal_	0.22			0.56			-		
R^2^_conditional_	0.95			0.95			-		
AIC	1268			1116			-		

Coef: coefficient, SE: standard error, CI: confidence interval, TEMP: temperature, MO: match outcome, ML: match location, OQ: opponent quality, BP: ball in play, RC: red cards, AIC: Akaike information criterion; * denotes inadequate model fit.

**Table 3:** The effect of ambient temperature on the distance covered at higher speeds while controlling for situational factors.

	High-Speed Running (Model 4)			Sprinting (Model 5)			High-Intensity Running (Model 6)		
Fixed Effects	Coef (SE)	95%CI	Effect Size	Coef (SE)	95% CI	Effect Size	Coef (SE)	95%CI	Effect Size
Intercept	6.37 (0.13)	6.11–6.64		2.24 (0.07)	2.11–2.38		8.56 (0.19)	8.19–8.94	
TEMP: ≤5 °C	1.03 (0.32)	0.39–1.67	0.62 (0.23–1.01)	0.33 (0.18)	−0.04–0.7	0.34 (−0.04–0.73)	1.36 (0.46)	0.44–2.28	0.57 (0.18–0.96)
TEMP: 6–10 °C	0.92 (0.2)	0.52–1.32	0.89 (0.49–1.29)	0.27 (0.12)	0.05–0.5	0.22 (0.04–0.4)	1.2 (0.29)	0.62–1.77	0.8 (0.4–1.19)
TEMP: 11–20 °C	0.35 (0.13)	0.1–0.61	0.54 (0.15–0.93)	0.08 (0.07)	−0.07–0.22	0.2 (−0.18–0.58)	0.43 (0.18)	0.07–0.8	0.45 (0.07–0.84)
MO: Loss	-	-	-	-	-	-	-	-	-
MO: Draw	-	-	-	-	-	-	-	-	-
ML: Home	-	-	-	0.1 (0.02)	0.06–0.15	0.93 (0.52–1.34)	0.21 (0.06)	0.1–0.32	0.74 (0.33–1.15)
OQ	-	-	-	-	-		-	-	-
BP	-	-	-	-	-		-	-	-
RC: No	-	-	-	-	-		-	-	-
Random effects	Coef (SE)	95%CI	*p*	Coef (SE)	95%CI	*p*	Coef (SE)	95%CI	*p*
Match	0.28 (0.05)	0.19–0.4	<0.001	0.1 (0.02)	0.08–0.14	<0.001	0.19 (0.03)	0.14–0.25	<0.001
Team	0.23 (0.07)	0.13–0.41	0.001	0.03 (0.01)	0.02–0.06	0.002	0.65 (0.11)	0.47–0.89	0.001
Residuals	0.14 (0.02)	0.11–0.19	<0.001	0.03 (0)	0.02–0.04	<0.001	0.36 (0.11)	0.2–0.66	<0.001
R^2^_marginal_	0.09			0.08			0.10		
R^2^_conditional_	0.90			0.92			0.94		
AIC	487			157			614		

Coef: coefficient, SE: standard error, CI: confidence interval, TEMP: temperature, MO: match outcome, ML: match location, OQ: opponent quality, BP: ball in play, RC: red cards, AIC: Akaike information criterion.

## Data Availability

The raw data supporting the conclusions of this article will be made available by the authors on request.
